# Defective Awakening Response to Nocturnal Hypoglycemia in Patients with Type 1 Diabetes Mellitus

**DOI:** 10.1371/journal.pmed.0040069

**Published:** 2007-02-27

**Authors:** Bernd Schultes, Kamila Jauch-Chara, Steffen Gais, Manfred Hallschmid, Eva Reiprich, Werner Kern, Kerstin M Oltmanns, Achim Peters, Horst L Fehm, Jan Born

**Affiliations:** 1 Department of Internal Medicine, University of Luebeck, Luebeck, Germany; 2 Department of Neuroendocrinology, University of Luebeck, Luebeck, Germany; 3 Department of Psychiatry and Psychotherapy, University of Luebeck, Luebeck, Germany; Lund University Hospital, Sweden

## Abstract

**Background:**

Nocturnal hypoglycemia frequently occurs in patients with type 1 diabetes mellitus (T1DM). It can be fatal and is believed to promote the development of the hypoglycemia-unawareness syndrome. Whether hypoglycemia normally provokes awakening from sleep in individuals who do not have diabetes, and whether this awakening response is impaired in T1DM patients, is unknown.

**Methods and Findings:**

We tested two groups of 16 T1DM patients and 16 healthy control participants, respectively, with comparable distributions of gender, age, and body mass index. In one night, a linear fall in plasma glucose to nadir levels of 2.2 mmol/l was induced by infusing insulin over a 1-h period starting as soon as polysomnographic recordings indicated that stage 2 sleep had been reached. In another night (control), euglycemia was maintained.

Only one of the 16 T1DM patients, as compared to ten healthy control participants, awakened upon hypoglycemia (*p* = 0.001). In the control nights, none of the study participants in either of the two groups awakened during the corresponding time. Awakening during hypoglycemia was associated with increased hormonal counterregulation. In all the study participants (from both groups) who woke up, and in five of the study participants who did not awaken (three T1DM patients and two healthy control participants), plasma epinephrine concentration increased with hypoglycemia by at least 100% (*p* < 0.001). A temporal pattern was revealed such that increases in epinephrine in all participants who awakened started always before polysomnographic signs of wakefulness (mean ± standard error of the mean: 7.5 ± 1.6 min).

**Conclusions:**

A fall in plasma glucose to 2.2 mmol/l provokes an awakening response in most healthy control participants, but this response is impaired in T1DM patients. The counterregulatory increase in plasma epinephrine that we observed to precede awakening suggests that awakening forms part of a central nervous system response launched in parallel with hormonal counterregulation. Failure to awaken increases the risk for T1DM patients to suffer prolonged and potentially fatal hypoglycemia.

## Abstract 2

A study of 16 patients with type 1 diabetes and 16 healthy participants showed that the normal awakening response provoked by a fall in plasma glucose was impaired in diabetic individuals.

## Editors' Summary

**Background.:**

Hypoglycemia (low blood sugar) is a frequent complication of insulin-treated diabetes, affecting patients with type 1 diabetes mellitus in particular. In individuals who do not have diabetes, insulin secretion is modified naturally and continuously by the body's own regulatory systems, depending on the blood sugar. However, in diabetes patients there is a lack of natural insulin and so manufactured insulin has to be given by injection after blood sugar testing. Hence, it is not possible for patients with diabetes to modify insulin secretion naturally in response to a change in glucose levels, and so blood glucose levels can rise and fall beyond healthy levels. In individuals who have intensive insulin therapy, hypoglycemia can be a particular problem; each year about 25% of patients on intensive insulin therapy have at least one episode of severe hypoglycemia—which requires the assistance of another person.

When hypoglycemia occurs during the day, diabetes patients can recognize it by a variety of symptoms, e.g., feeling sweaty and lightheaded, and they may either seek help from another person or treat themselves with sugar. Hypoglycemia during sleep may be very common—it has been observed to occur in up to half of the nights when patients with diabetes were monitored. The particular problem with hypoglycemia occurring during sleep is that diabetes patients may not be aware of it and hence may not be able to treat themselves or to seek assistance. It is believed to contribute to some instances of sudden death during sleep in patients with diabetes.

**Why Was This Study Done?:**

It has not been clear whether there is a certain level of blood glucose below which a signal is triggered that provokes awakening from sleep in either diabetes patients or in individuals who do not have diabetes. The authors of this study wanted to compare responses to lowered blood glucose in diabetes patients and in individuals who do not have diabetes and to see whether the responses differed. They also wanted to look at whether there were any other hormonal changes that preceded or followed awakening after hypoglycemia.

**What Did the Researchers Do and Find?:**

They treated two groups; 16 type 1 diabetes mellitus patients and 16 healthy control participants. With careful monitoring, on one night once stage 2 sleep (as measured by a method known as polysomnography) had been reached, they gave insulin to lower the blood glucose to a specific level (2.2 mmol/l), which would when awake give symptoms of hypoglycemia. On another night (the control night) normal blood sugar levels were maintained.

They found that only one of the 16 diabetes patients, as compared to ten healthy control participants, woke when hypoglycemia occurred. In the control nights, none of the study participants in either of the two groups awakened during the corresponding time. Awakening during hypoglycemia was associated with substantial hormonal changes, especially with an increase in one hormone, epinephrine (also known as adrenaline), and the increases in this hormone occurred before polysomnographic signs of wakefulness.

**What Do These Findings Mean?:**

It appears that patients with type 1 diabetes mellitus do not awake at a level of hypoglycemia that triggers waking in normal individuals. The hormonal responses that were seen in individuals who awoke may be part of a crucial response system to hypoglycemia. These results help us to understand how diabetes patients respond to hypoglycemia, but further work will need to be done to determine whether it is possible to improve the response. It should be noted, however, that the results are probably not generalizable to patients with type 2 diabetes mellitus, who represent the majority of patients with diabetes.

**Additional Information.:**

Please access these Web sites via the online version of this summary at http://dx.doi.org/10.1371/journal.pmed.0040069.

- A related *PLoS Medicine* Perspective article by Ilan Gabriely and Harry Shamoon discusses this study and more about hypoglycemia in T1DM
- MedlinePlus, the encyclopedia of health information from the US National Library of Medicine, has a collection of pages on hypoglycemia
- Wikipedia pages on hypoglycemia (note that Wikipedia is a free online encyclopedia that anyone can edit)
- National Diabetes Information Clearinghouse, a service of the US National Institute of Diabetes and Digestive and Kidney Diseases, has information on hypoglycemia

## Introduction

Hypoglycemia is the limiting factor in the therapeutic management of type 1 diabetes mellitus (T1DM) [1]. Each year, about 25% of T1DM patients with intensive insulin therapy experience at least one episode of severe hypoglycemia defined by a hypoglycemic state requiring assistance of another person [2,3]. It has been estimated that about 55% of severe hypoglycemic episodes occur during sleep [4]. Mild to moderate hypoglycemic episodes lasting between 1 h and 12 h have been observed in 27%–56% of monitored nights in T1DM patients [5–12]. Although these episodes appear to be asymptomatic most of the time [7,8,11], hypoglycemic episodes during sleep constitute a particular problem in the management of T1DM, since even a single hypoglycemic episode can reduce awareness of, and neuroendocrine counterregulation against, subsequent hypoglycemia [13,14]. Hence, undetected hypoglycemic episodes during sleep may substantially contribute to the development of a hypoglycemia-unawareness syndrome frequently encountered in T1DM patients [15]. Moreover, undetected nocturnal hypoglycemia has been considered to be responsible for a large proportion of sudden deaths in young T1DM patients, i.e., the dead-in-bed syndrome [16]. Such observations suggest that hypoglycemia does not induce proper awakening in these patients, thereby preventing an adequate behavioral control of this potentially dangerous state. However, whether hypoglycemia normally induces awakening in individuals who do not have diabetes, but does not do so in T1DM patients, is at present unclear.

Jones et al. [17] have previously shown that neuroendocrine counterregulation against hypoglycemia is impaired during sleep in both T1DM patients and in healthy adolescents, which could partly explain a tendency toward prolonged nocturnal hypoglycemic episodes in the T1DM patients. In healthy adults, sleep increased the glycemic threshold for neuroendocrine counterregulatory responses, which means that responses started at a lower plasma glucose level during sleep than during wakefulness [18]. In this latter study, the onset of the counterregulatory response coincided with a transition to lighter sleep, with most healthy control participants showing signs of awakening.

The effects of hypoglycemia on sleep in T1DM patients have rarely been assessed systematically. Spontaneous hypoglycemia of variable extent in children with T1DM was found to be associated with increased slow-wave sleep [19,20]. However, these studies leave open the question of whether deepening of sleep is a cause or an effect of hypoglycemia. There are also many clinical observations suggesting that adult T1DM patients do not awaken during hypoglycemia [11,12,21]. Hypoglycemia is more likely to cause seizures at night than during daytime [22] and, in sleeping adult T1DM patients, electroencephalogram abnormalities were identified at an increased frequency during spontaneous hypoglycemic episodes with blood glucose levels of less than 2.2 mmol/l [5,23]. Such alterations point to a specific difficulty to awake during a hypoglycemic state in these patients.

A recent study investigated T1DM patients and healthy control participants during stepwise hypoglycemic clamps performed in the late evening [24]. When the T1DM patients were allowed to fall asleep after 2.5 h on the clamp, they exhibited distinctly greater amounts of sleep than did the healthy control participants during the subsequent 2.5 h on the clamp, with this dissociation attaining significance when the lowest hypoglycemic level of 2.5 mmol/l was reached. These data indicate an increased ability in T1DM patients to fall asleep during a moderate hypoglycemia that was initiated some time before sleep.

In the present study, we aimed to answer the question as to whether hypoglycemia that emerges during ongoing sleep, and the associated fall in blood glucose concentrations, triggers a signal that, at a certain level, provokes awakening from sleep in these patients. Awakening responses were compared between T1DM patients and healthy control participants. To elucidate whether awakening is a prerequisite for a counterregulatory hormonal response, we also examined whether awakening preceded or followed the hormonal response to hypoglycemia.

## Methods

### Participants

We studied 16 T1DM patients (men/women: 9/7) and 16 healthy control participants (8/8) of comparable age (mean ± standard error of the mean: 31.3 ± 2.6 y and 28.4 ± 1.5 y) and body mass index (24.4 ± 0.8 kg/m^2^ and 23.0 ± 0.6 kg/m^2^). Patients with T1DM were selected to participate only when they had no clinical evidence of an autonomic neuropathy or any other complication affecting patients with diabetes. Mean diabetes duration in the patients was 9.1 ± 1.4 y (range: 1–23 y). Mean HbA1c was 7.7% ± 0.3% (range: 6.0%–10.0%; upper limit of the normal range: 6.8%). None of the T1DM patients or control participants received any medication at the time of the experiments except for insulin therapy in the T1DM patients. Twelve patients were on an intensive conventional therapy regime with at least three injections of regular insulin and between one and two injections of long-acting insulin per day. The remaining four T1DM patients were on continuous subcutaneous insulin infusion. The mean ± standard error of the mean cumulative insulin dose was 55.8 ± 3.8 U per day (range: 37–82 U).

All T1DM patients measured their blood glucose concentration at least four times per day. Eleven patients reported regularly feeling hypoglycemic at threshold levels above 2.8 mmol/l, whereas the remaining five T1DM patients reported noticing hypoglycemia only at levels of less than 2.8 mmol/l. Based on this clinical criterion, these five patients were classified as displaying an impaired awareness of hypoglycemia. Only three of the 16 T1DM patients had reported experiencing an episode of severe hypoglycemia within the past year, i.e., being in a hypoglycemic state and requiring assistance by another person. Nine patients reported having had at least one such episode since being diagnosed with diabetes.

The study conformed to the Declaration of Helsinki and was approved by the Ethics Committee on Research Involving Humans at the University of Luebeck. All study participants gave written informed consent.

### Experimental Protocol

Following an adaptation night in the laboratory, including the placement of intravenous catheters and electrodes for standard polysomnography, each patient with T1DM and each control participant was tested during two experimental nights, spaced at least 2 wk apart. In one night, hypoglycemia was induced by intravenous insulin infusion (hypoglycemic night). In the other night (control), euglycemic conditions (>3.9 mmol/l) were maintained and plasma glucose concentrations were measured hourly. Spontaneous hypoglycemia was prevented by glucose infusion when necessary—and this occurred in five of the T1DM patients' control nights. The order of experimental conditions was balanced across both the T1DM patients and the control participants. In other words, eight T1DM patients and eight healthy control participants started with the hypoglycemic condition, while the remaining participants first performed the control condition. Within this scheme, each T1DM patient and control participant was randomly allotted his or her individual order. All T1DM patients and control participants were informed that they could be subjected to short periods of hypoglycemia during one night, but they did not know whether and when this occurred. Thus, experiments were performed in a single-blind fashion. T1DM patients were instructed to inject their regular dose of long-acting insulin before going to bed or to keep their insulin pump at the regular infusion rate throughout the night.

### Procedure

On each experimental night, T1DM patients and control participants reported to the laboratory at 2000 hours. Two intravenous catheters were inserted in the distal part of the right and left forearms for blood sampling and infusion of insulin (Insuman Rapid, Aventis, http://www.sanofi-aventis.de). Electrodes were attached to the scalp (electroencephalogram), around the eyes (horizontal and vertical electrooculogram), and on the chin (electromyogram) for standard polysomnography. Both groups of study participants went to bed and lights were turned off at 2300 hours.

The polysomnographic recordings were monitored online for signs of sleep. In the hypoglycemic night, insulin infusion started at a continuous rate of 1.5 mU per kg of body weight per min after the T1DM patient or control participant had reached sleep stage 2 for the first time and had remained in this or a deeper sleep stage for 3 min. Insulin was infused for about 60 min while plasma glucose was measured at 5-min intervals using the Glucose Analyzer II (Beckman Instruments, http://www.beckmancoulter.com). Plasma glucose was allowed to fall in a linear manner such that a target nadir level of 2.2 mmol/l was reached after ∼60 min. To control the temporal dynamics of the decrease in glucose concentration, a 20% glucose solution was infused concurrently whenever necessary. At the nadir concentration of 2.2 mmol/l, insulin infusion was stopped and plasma glucose levels were brought back into the normal range by glucose infusion. A nadir level of 2.2 mmol/l was selected based on available data which indicated that, at this level, hypoglycemia can be safely induced without exposing the T1DM patient or control participant to the risk of seizure or any other major adverse events.

During the 60-min period of insulin infusion, blood samples were collected every 5 min for determining counterregulatory hormone concentrations. Blood sampling and infusions were carried out via long thin tubes (dead space: 1.5 ml) from an adjacent room without disturbing the individual's sleep. Samples used for analysis were collected after drawing a buffer volume of about 5 ml.

### Measurements

Polysomnographic recordings were scored offline according to standard criteria [25] by two experts who were not aware of whether the recordings were taken during the hypoglycemia night or the control night. The following sleep parameters were determined for the whole night: sleep period time (time from sleep onset until final awakening defined by the last occurrence of any sleep stage except “awake”); total sleep time (time spent in sleep stages 1, 2, 3, and 4, and rapid eye movement [REM] sleep between sleep onset and final awakening); time spent awake after sleep onset in sleep stages 1, 2, 3, and 4; time spent in slow-wave sleep (i.e., sleep stages 3 and 4); REM sleep (in minutes and percentage of sleep period time); and latency of the first occurrence of slow-wave sleep and REM sleep (with reference to sleep onset). In addition, time spent in the different sleep stages during the final 10-min epoch of insulin infusion and during the corresponding interval of the control nights was determined in order to reveal acute effects of hypoglycemia. The target variable of the study was the occurrence of awakening during the hypoglycemic interval in T1DM patients and control participants, as defined by polysomnographic criteria. Although clinically relevant, we did not confirm the individual's awakening and responsiveness (e.g., by asking him/her if he/she is awake) in order not to interrupt the experiment. The definition of wakefulness according to standard polysomnographic criteria includes, amongst others, brief muscle arousal, an increase in electromyographic activity, and a change to low-amplitude mixed-frequency electroencephalogram activity with increased proportions of alpha-waves (8–13 Hz) [25].

The following assays were used to measure counterregulatory hormonal responses. Plasma epinephrine and norepinephrine were assessed using standard high-pressure liquid chromatography; inter-assay coefficient of variation (CV) and intra-assay CV were, respectively, epinephrine <5.6% and <2.9% and norepinephrine <6.1% and <2.6%. Plasma ACTH was measured using luminescence immunoassay (Lumitest, Brahms Diagnostica, http://www.brahms.de), with inter-assay CV <5.1% and intra-assay CV <3.2%. Serum cortisol was assessed using enzyme immunoassay (Enzymun-Test Cortisol, Roche Diagnostics, http://www.roche.com), with inter-assay CV <3.9% and intra-assay CV <2.0%. Serum growth hormone was measured using radioimmunoassay (HGH, Diagnostic Products, http://www.dpcweb.com), with inter-assay CV <3.4% and intra-assay CV <1.6%. Plasma glucagon was assessed using radioimmunoassay (Serono Diagnostics, http://www.serono.com), with inter-assay CV <6.1% and intra-assay CV <4.9%. Serum insulin concentrations were assessed using ELISAs (Dako Cytomation, http://www.dako.com), with inter-assay CV 7.5% and intra-assay CV 6.7%.

### Statistical Analysis

Values are presented as mean ± standard error of the mean. Pairwise comparisons of the frequency of awakening and of above-threshold counterregulatory hormonal responses between groups were based on chi-square tests. Sleep data were analyzed by Student's *t*-test and, where appropriate, using nonparametric tests (Mann-Whitney U-test and Wilcoxon signed rank test). Hormonal parameters were analyzed by analysis of variance (ANOVA) with the repeated-measures factor “time” representing the repeated hormonal assessments. For differences between T1DM patients and control participants, a “group” factor was included, with the “group × time” interaction term indicating different hormonal profiles over time. Note that since we did not apply a formal adjustment of tests for multiple comparisons (as such an adjustment did not appear to be fully appropriate here), the statistical outcomes generally should be interpreted with caution. Above-threshold counterregulatory hormonal responses were defined separately for each hormone. Threshold levels for epinephrine, ACTH, cortisol, and growth hormone were defined as an increase of 100% with reference to the preceding 5-min interval. Norepinephrine and glucagon threshold levels were defined by a respective increase of more than 50% within 5 min. The choice of threshold criteria was based on previous studies which proved that these criteria enabled identification of an emerging counterregulatory hormonal response during stepwise hypoglycemic clamp experiments, unconfounded by assay variability or by spontaneous hormonal fluctuations. The point in time at which levels exceeded the threshold for the first time was defined as the onset of the respective counterregulatory response. Degrees of freedom were corrected according to the Greenhouse-Geisser procedure where necessary. A *p*-value of less than 0.05 was considered significant.

## Results

### Plasma Glucose and Sleep Recordings

**Figure 1:**
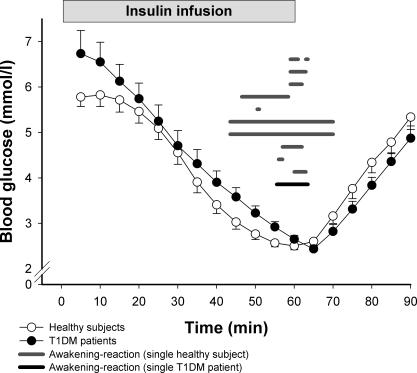
Plasma Glucose Concentration during Insulin-Induced Hypoglycemia Mean ± standard error of the mean plasma glucose concentration in 16 control participants (open circles) and in 16 patients with T1DM (filled circles) during insulin-induced hypoglycemia and the subsequent 30 min. Grey horizontal bars indicate intervals of wakefulness (determined by polysomnography) in ten out of 16 healthy individuals awakening upon hypoglycemia. The black horizontal line represents time awake for the only T1DM patient who awoke during hypoglycemia. There were no differences (*p* > 0.45) between T1DM patients and healthy control participants in glucose infusion rates during the 1-h hypoglycemic interval or between the study participants (from either group) who did and did not wake up.

Before infusion of insulin, plasma glucose concentration averaged 6.7 ± 0.5 mmol/l in the T1DM patients and 5.8 ± 0.3 mmol/l in the control participants (*p =* 0.106). Serum insulin levels were higher in the T1DM patients than in the control participants (138 ± 24 pmol/l versus 66 ± 12 pmol/l; *p* = 0.012). During the insulin infusion, serum insulin levels markedly increased but did not differ between the two groups (1,110 ± 162 pmol/l versus 1,014 ± 120 pmol/l; *p* = 0.630). The insulin infusion started as soon as the T1DM patient's or control participant's polysomnographic recordings indicated sleep stage 2 or deeper sleep for 3 min, which was between 23:05 and 00:45 h. On average, infusion of insulin started at 23:44 h in the T1DM patients and at 23:36 h in the control participants. The following decrease in plasma glucose concentration as well as its temporal characteristics was similar between the groups (Figure 1). The nadir plasma glucose concentration in the T1DM patients reached 2.22 ± 0.01 mmol/l, and 2.24 ± 0.02 mmol/l in the healthy control participants (*p =* 0.982).

During decreasing plasma glucose levels, ten of the 16 control participants showed clear-cut polysomnographic signs of awakening, whereas this was the case in only one of the 16 T1DM patients (*p =* 0.001). During the corresponding time interval (i.e., the hour after the first occurrence of sleep stage 2) in the control nights, in which no hypoglycemia was induced, spontaneous awakening was not observed in any of the T1DM patients or control participants. This result indicates that the increased number of awakenings observed during the hypoglycemia in the control participants reflects a response to decreasing plasma glucose levels. The glucose concentrations at which the ten control participants awakened ranged between 2.2 and 2.7 mmol/l (mean ± standard error of the mean: 2.3 ± 0.1 mmol/l). The one T1DM patient who awakened did so at a concentration of 2.7 mmol/l.

**Figure 2:**
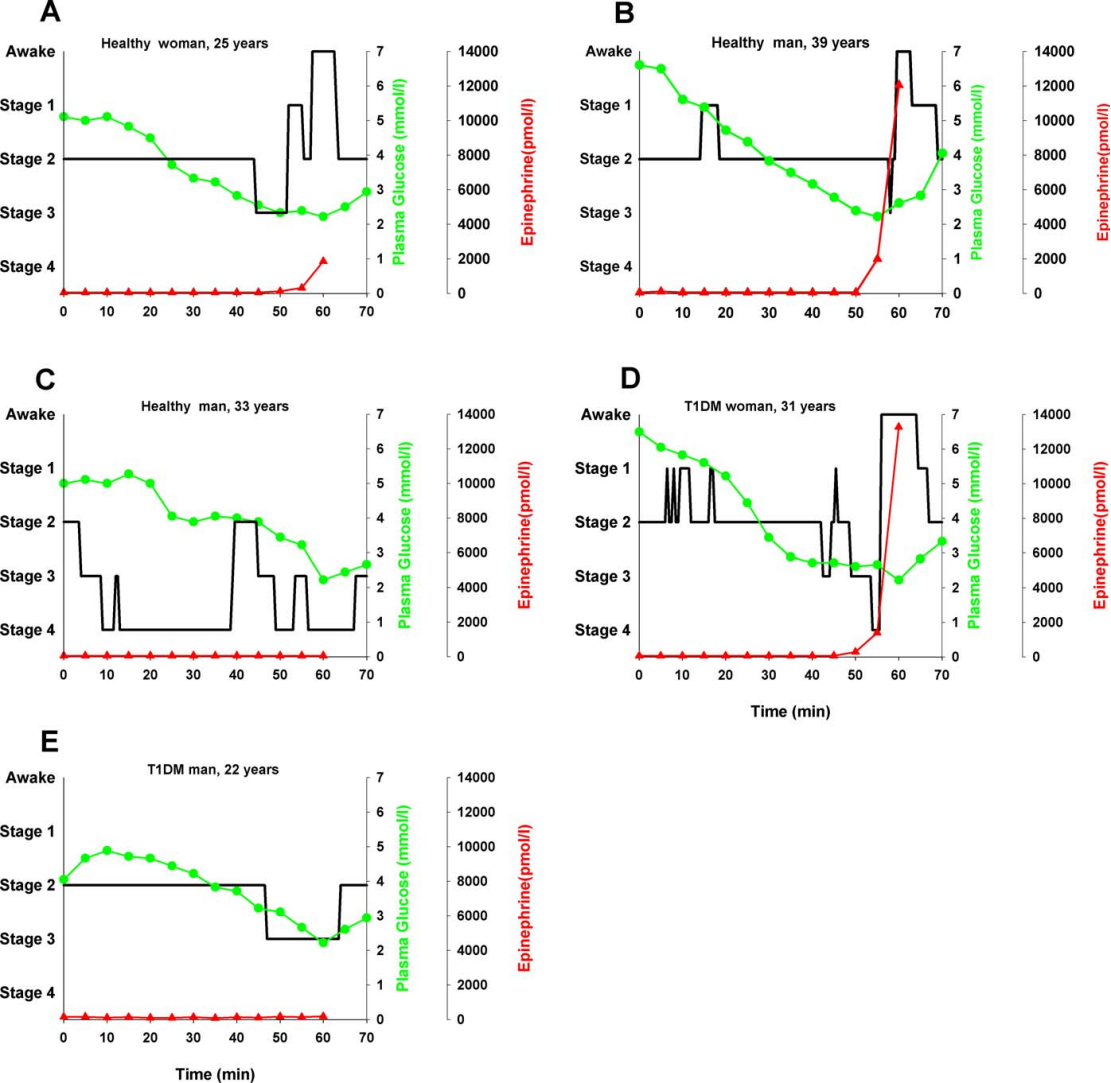
Sleep, Plasma Glucose, and Epinephrine Concentrations in Five Individuals during Insulin-Induced Hypoglycemia The panels depict time courses of polysomnographically recorded sleep (black line) in parallel with plasma glucose (green line) and epinephrine (red line) concentrations during the 1-h insulin infusion (0 to 60 min). (A and B) Data from two healthy control participants who displayed the typical awakening response to hypoglycemia. (C) Data from a healthy control participant who did not awaken during hypoglycemia and remained in slow-wave sleep (sleep stages 3 and 4). (D) Data from the only one of the 16 T1DM patients who awakened during hypoglycemia. (E) Data from a typical T1DM patient who did not awaken during hypoglycemia. Note that all study participants who woke up (A, B, and D) show marked rises in epinephrine levels that always start before awakening. Note also that awakening occurred fairly abruptly in most cases (e.g., B and D), and was not preceded by a gradual lightening of sleep. (Left *x*-axis refers to sleep stages—awake and sleep stages 1 to 4).

Figure 2 illustrates representative time courses of polysomnographic sleep together with plasma glucose concentrations during infusion of insulin in different individuals. In most T1DM patients or control participants who awoke during hypoglycemia, this awakening occurred abruptly from slow-wave sleep without a preceding period of more shallow sleep.

The differential awakening response to hypoglycemia between T1DM patients and healthy control participants was confirmed in an analysis of the sleep structure focusing on the 10-min interval preceding the time of nadir glucose concentration (summarized in Table 1). Mean proportion of time awake during the 10 min before nadir glucose concentration was 16% in the control participants and 3% in the T1DM patients (*p* = 0.005). Analyses of the total night, as expected, revealed less total sleep in the hypoglycemic than in the control nights. However, this difference was not significant, as the effects were diluted by the remaining hours of uninterrupted sleep.

**Table 1:**
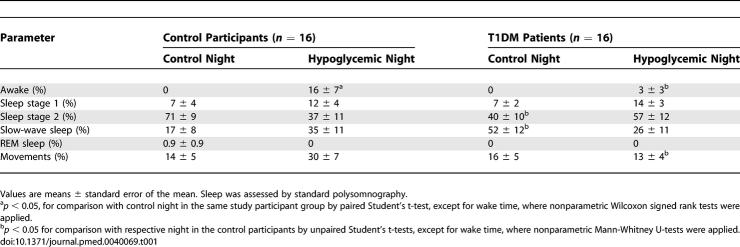
Sleep Parameters during the Final Ten Minutes Preceding the Nadir of Plasma Glucose on the Hypoglycemic Nights and during the Corresponding Time Intervals on the Control Nights

### Counterregulatory Hormones

**Figure 3:**
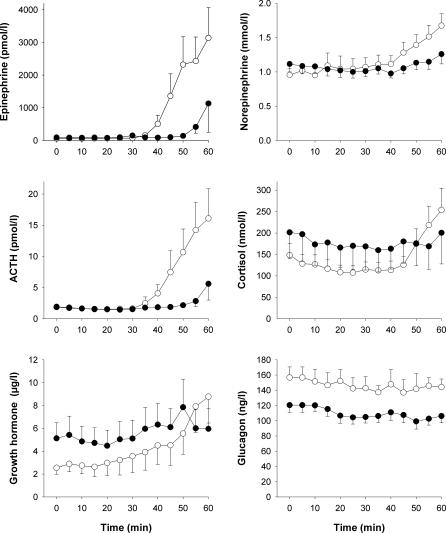
Counterregulatory Hormonal Responses during Insulin-Induced Hypoglycemia Mean ± standard error of the mean counterregulatory hormone concentrations during insulin-induced hypoglycemia in the 16 healthy control participants (open circles) and in the 16 T1DM patients (filled circles).

In parallel with the awakening response, an above-threshold response of epinephrine (*p* = 0.005) and ACTH (*p* = 0.003) to hypoglycemia was observed distinctly more often in the control participants than in the T1DM patients (Table 2). As depicted in Figure 3, control participants responded to hypoglycemia with strong increases in mean concentrations of epinephrine (*p* = 0.005, for the ANOVA time factor), norepinephrine (*p* < 0.001), ACTH (*p* = 0.009), cortisol (*p* = 0.015), and growth hormone (*p* = 0.012). In contrast, in the T1DM patients, these responses were weaker and not significant (*p =* 0.278, *p =* 0.367, *p =* 0.166, *p =* 0.572, and *p =* 0.740, respectively). ANOVA confirmed differences in hormonal responses to hypoglycemia between the groups for epinephrine (*p* < 0.001, for the ANOVA “group × time” interaction term), norepinephrine (*p* = 0.006), ACTH (*p* = 0.037), and cortisol (*p* = 0.001). The differences in the responses between groups for growth hormone were not significant (*p* = 0.084). Glucagon concentrations remained unchanged by hypoglycemia in both healthy control participants (*p* = 0.153) and T1DM patients (*p* = 0.842). Overall, glucagon levels were lower in the T1DM patients than in the control participants (*p* = 0.025).

**Table 2:**
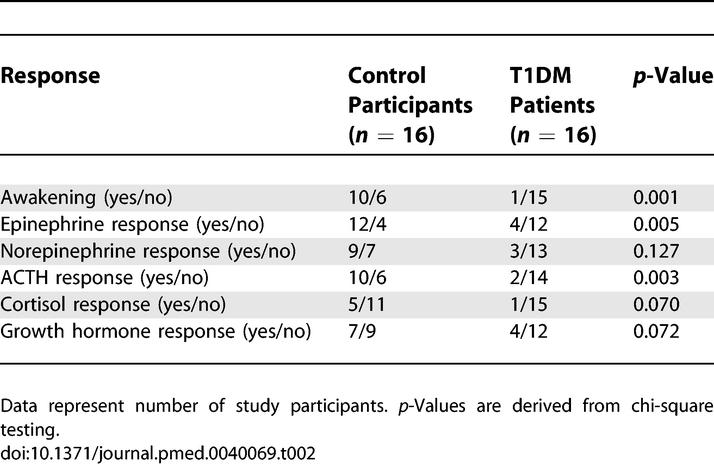
Comparison of Awakening and Above-Threshold Endocrine Responses to Nocturnal Hypoglycemia between Healthy Control Participants and T1DM Patients

### Comparison of T1DM Patients and Control Participants with and without Awakening Response

Grouping study participants with respect to the presence versus the absence of an awakening response to hypoglycemia (across both the T1DM patients and the control participants) revealed that awakening was associated with a strikingly increased likelihood of an above-threshold response to hypoglycemia in concentrations of epinephrine (*p <* 0.001), ACTH (*p <* 0.001), and cortisol (*p =* 0.005). However, an increase in epinephrine levels during hypoglycemia was also observed in five study participants (three T1DM patients and two healthy control participants) who did not wake up, indicating that an awakening response is not a prerequisite for the activation of neuroendocrine counterregulation**.** On the other hand, all T1DM patients and control participants awakening upon hypoglycemia showed a clear-cut increase in epinephrine levels, and this rise started in all cases before polysomnographic signs of awakening occurred (on average 7.5 ± 1.6 min; range: 2–17 min).

There were no differences in age (28.4 ± 2.2 y versus 28.3 ± 2.1 y; *p* = 0.984) and body mass index (22.6 ± 0.8 kg/m^2^ versus 23.8 ± 1.0 kg/m^2^; *p* = 0.359) between the healthy control participants who did and who did not wake up. With six out of eight men and four out of eight women showing an awakening response (*p* = 0.302), there was also no sign of a possible influence of gender.

## Discussion

Our data show that a decrease in plasma glucose concentration to a nadir of 2.2 mmol/l within 1 h during sleep induces a wake-up response in most healthy control participants. In contrast, in T1DM patients, the identical hypoglycemic stimulus very rarely causes awakening. The lack of an awakening response was paralleled by an absence of clear-cut counterregulatory hormonal responses to hypoglycemia in the great majority of T1DM patients.

Results from our healthy control participants provide evidence that hypoglycemia belongs to the biological class of stressors terminating sleep to allow adaptive behaviors such as food intake. Awakening in the control participants occurred at a plasma glucose level of less than 2.8 mmol/l (range: 2.2–2.7 mmol/l) and, in all these cases, the wake state persisted until glucose concentrations started to rise again. Given the low glucose levels at which awakening occurred, these findings agree with previous observations in healthy adolescents as well as in T1DM patients who did not show signs of awakening during a 40-min period of steady-state hypoglycemia at concentrations of between 2.8 and 3.1 mmol/l [17]. While the decrease in glucose was gradual, awakening in our control participants mostly occurred somewhat abruptly, not preceded by a transient period of lighter sleep. Such a pattern strongly speaks for the existence of a discrete glycemic threshold at which awakening regularly occurs. It should be pointed out that, for ethical reasons, the hypoglycemia induced in our study was only of short duration, and plasma glucose levels were not allowed to fall below 2.2 mmol/l. Assuming that in some individuals the threshold is at an even lower concentration, this can explain why not all of our control participants awoke around the time of their glucose nadir concentration. In combination, our data indicate the presence of a glycemic threshold of awakening that shows some variability among control participants, but is below 2.8 mmol/l in most individuals.

Since our goal was to determine thresholds for awakening, we induced hypoglycemia at a comparatively fast rate. This approach diverges from many previous studies that investigated thresholds for counterregulatory hormonal responses to hypoglycemia induced by a stepwise clamp procedure that extends over several hours. This procedure has been successfully applied to show that hypoglycemia (during a stepwise clamp procedure that was already initiated before sleep) leads to a general reduction in sleep efficiency [24]. However, the stepwise clamp procedure is less feasible for determining thresholds of discrete awakening responses because, with longer duration of hypoglycemic periods, there is an increased probability of confounding influences from spontaneous changes in sleep structure. Nevertheless, it should be noted that the awakening threshold is likely to be sensitive to rate effects so that, with a more gradual fall in glucose, thresholds might even be at a lower glucose concentration; however, this theory remains to be tested. Inducing a short-lived hypoglycemia at a comparatively rapid rate also prevents a straightforward comparison of the counterregulatory hormonal responses obtained here with those obtained in previous studies (e.g., [24]) that used slower, stepwise hypoglycemic clamps allowing examination of the fully developed counterregulatory response rather than—as in this study—a mere decision about the presence or absence of a hormonal response around the time of awakening. In order not to disturb the study participant's sleep by blood sampling, we measured plasma glucose in venous rather than, as in most previous studies, arterialized-venous blood. The venous blood measurements of the present study, however, provide higher glucose values than the measurements taken in previous studies and further hamper direct comparisons with those earlier studies.

It remains currently obscure as to what extent the awakening response to hypoglycemia is modulated by concurrent hyperinsulinemia. We did not perform hyperinsulinemic-euglycemic clamps during control nights to assess the effects of insulin during increased rates of glucose infusion. However, although indirect, evidence from previous studies using different modes of insulin and glucose administration does not point to any substantial effects of hyperinsulinemia itself on sleep (e.g., [26,27]). Importantly, since the rate of insulin infusion and the resulting hyperinsulinemia was identical in both T1DM patients and control participants, this factor cannot explain the difference in awakenings to hypoglycemia between the groups.

Since only one out of our 16 T1DM patients awoke during hypoglycemia, this precludes any statistically based characterization of patients showing and not showing a defective awakening response to hypoglycemia. Nevertheless, it should be pointed out that the one T1DM patient who woke up upon hypoglycemia, a 31-y-old woman (body mass index 21.1 kg/m^2^; Figure 2D), had the shortest diabetes duration (1 y). Thus, we speculate that an impaired awakening response upon hypoglycemia may develop with progressive duration of the disease. It is also noteworthy that although this woman awoke upon hypoglycemia, she exhibited several conditions that are well known to increase the risk for a syndrome of hypoglycemia unawareness—including counterregulatory failure. Thus, she had a comparatively low HbA1c level of 6.7%, which is slightly below the upper limit of the normal range (6.8%) and is also below the mean HbA1c level of the group of T1DM patients studied (7.7 ± 0.3%). In addition, she had previously experienced one episode of severe hypoglycemia (requiring a subcutaneous injection of glucagon by another person). With regard to the unawareness syndrome, our sample of T1DM patients overall was somewhat heterogeneous. Based on clinical criteria, five of the patients indicated tendencies toward the presence of this syndrome.

Awakening upon hypoglycemia was strongly associated with the emergence of counterregulatory hormonal responses. Importantly, in all cases, the marked increase in circulating epinephrine levels preceded the actual awakening, on average by 7.5 min. The rise in epinephrine was distinctly greater than that typically observed in conjunction with regular awakening in the morning [28]. Moreover, in five of the study participants (three T1DM patients and two healthy control participants), the epinephrine response was not followed by a polysomnographic wake state. This temporal pattern excludes the possibility that attaining wakefulness is essential for the emergence of a counterregulatory hormonal response. Rather, awakening upon hypoglycemia forms part of a central nervous system response that develops in parallel with counterregulatory hormonal responses, and presumably originates from hypothalamic influences on brainstem-arousing structures [29]. This view does not exclude the possibility that neuroendocrine and autonomic activity in response to hypoglycemia can facilitate awakening [30], although definitive conclusions on a causal relationship between hormonal changes and awakening cannot be drawn from our data.

Our results underscore an important, yet greatly neglected problem in the management of T1DM. In response to hypoglycemia, these patients do not only often fail to show a hormonal counterregulatory response, but also often fail to wake up when hypoglycemia develops during sleep. Lacking a proper awakening response, these patients have only a diminished chance to compensate behaviorally for sleep-associated hypoglycemia by ingesting carbohydrates. In turn, this leads to prolonged and aggravated nocturnal hypoglycemic episodes. Considering the potentially harmful effects of nocturnal hypoglycemia, which can even be fatal, restoring a proper awakening response to hypoglycemia is a therapeutic goal of utmost importance to these patients.

## Abbreviations

ANOVA: analysis of variance
CV: coefficient of variation
REM: rapid eye movement
T1DM: type 1 diabetes mellitus
